# Aortic dissection induced by vascular endothelial growth factor inhibitors

**DOI:** 10.3389/fphar.2023.1189910

**Published:** 2023-06-22

**Authors:** Shuqi Dai, Yu Zhong, Hongxia Cui, Jin Zhao, Su Li

**Affiliations:** Department of Pharmacy, Cancer Hospital of China Medical University, Liaoning Cancer Hospital & Institute, Shenyang, China

**Keywords:** vascular endothelial growth factor inhibitors, adverse reaction, aortic dissection, hypertension, drug safety

## Abstract

Vascular endothelial growth factor (VEGF) contributes to angiogenesis and vasculogenesis. The occurrence and progression of tumors are accompanied by angiogenesis. Vascular endothelial growth factor inhibitors (VEGFI) have been used in anti-tumor treatment. However, aortic dissection (AD) is one of the VEGFI-associated adverse reactions with cute onset, rapid progression, and high case fatality rate. We collected case reports of VEGFI related to aortic dissection in PubMed and CNKI (China National Knowledge Infrastructure) from inception to 28 April 2022. Seventeen case reports were selected. The medication included sunitinib, sorafenib, pazopanib, axitinib, apatinib, anlotinib, bevacizumab, and ramucirumab. This review discusses the pathology, risk factors, diagnosis, and treatment of AD. Vascular endothelial growth factor inhibitors are related to aortic dissection. Although current literature lacks clear statistical evidence on the population, we offer points to encourage further confirmation of the best methods of care for these patients.

## 1 Introduction

**FIGURE 1:**
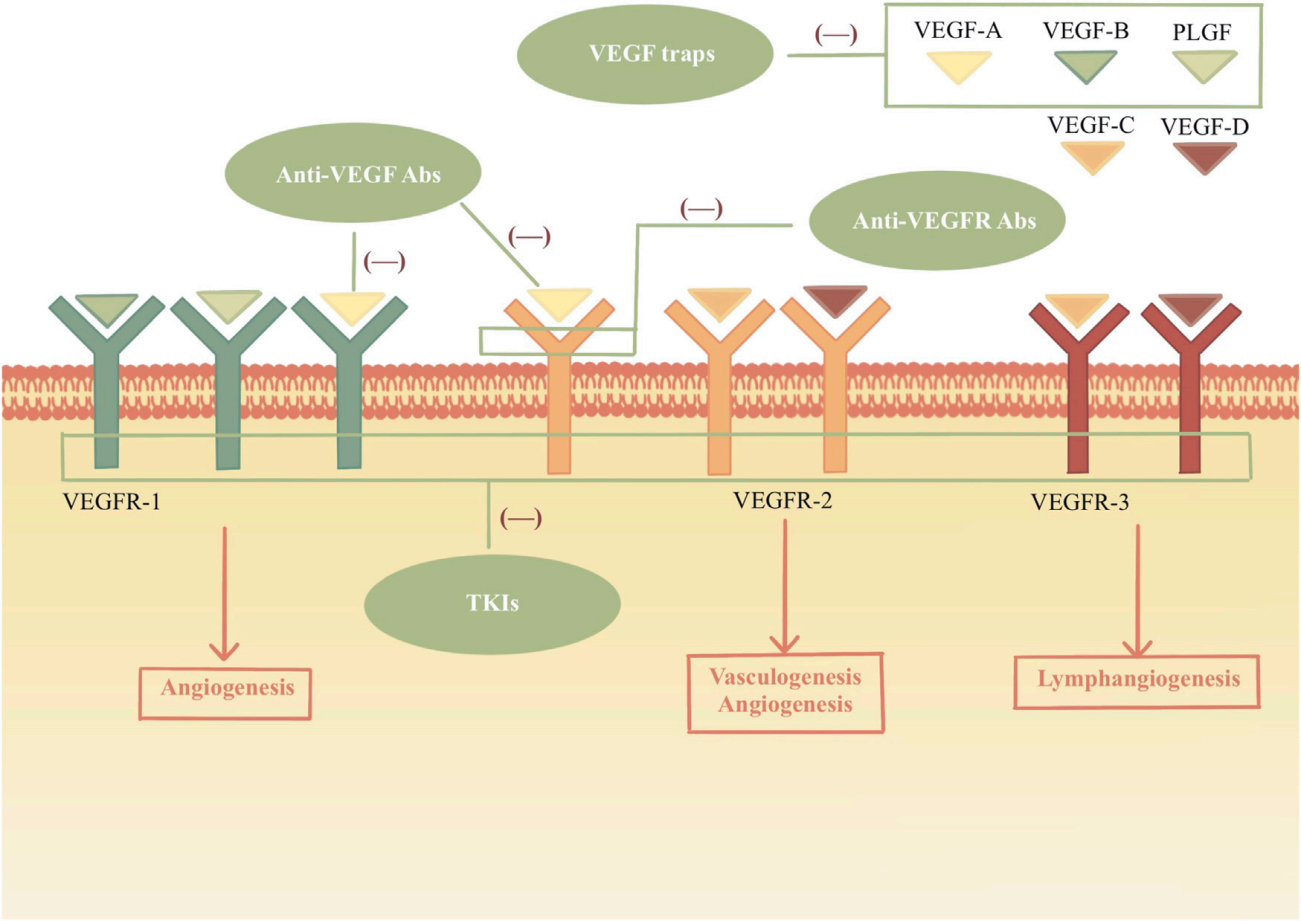
Mechanism of VEGF and inhibitors. VEGF family bind to the VEGF receptors and tyrosine kinases are activated, which leads to the angiogenesis, vasculogenesis and lymphangiogenesis. VEGFIs inhibit the VEGF signal pathways by acting different sites. Anti-VEGF Abs: anti-VEGF monoclonal antibodies; Anti-VEGFR Abs: anti-VEGFR monoclonal antibodies; TKIs: tyrosine kinase inhibitors.

Tumor cells secrete vascular endothelial growth factor (VEGF) promoting neovascularization to provide the oxygen and nutrients for tumor growth. VEGF and its receptor signaling pathway are important in angiogenesis. Anti-angiogenesis drugs mainly target VEGF and VEGF receptors (VEGFR) by inhibiting their expression, which blocks the signal transduction pathway or exhausts the VEGF produced by tumor cells (Figure 1). Therefore, they inhibit the generation of neovascularization and suspend the blood supply for tumors, restraining tumor growth, development, and metastasis.

Four classes of VEGF inhibitors (VEGFI) have been developed (Table 1): (a) anti-VEGF monoclonal antibodies; (b) anti-VEGFR monoclonal antibodies; (c) VEGF traps; (d) tyrosine kinase inhibitors (TKIs) (Dobbin et al., 2021).

**TABLE 1:** The classification and targets of the VEGFI.

Type	Drug	Target
anti-VEGF monoclonal antibodies	Bevacizumab	VEGF-A
anti-VEGFR monoclonal antibodies	Ramucirumab	VEGFR-2
VEGF traps	Aflibercept	VEGF-A, VEGF-B, PLGF
tyrosine kinase inhibitors (TKIs)	Sunitinib	VEGFR-1, VEGFR-2, VEGFR-3, KIT, FLT3, CSF1R, PDGFRA, PDGFRB, MET
	Pazopatib	VEGFR-1, VEGFR-2, VEGFR-3, PDGFRA, PDGFRB, KIT, FGFR3, ITK, FGF1, SH2B3
	Axitinib	VEGFR-1, VEGFR-2, VEGFR-3, PDGFRB, KIT, FLT3
	Cabozatinib	VEGFR-2, MET, RET
	Cediranib	VEGFR-1, VEGFR-2, VEGFR-3, PDGFR, KIT, FLT3
	Lenvatinib	VEGFR-1, VEGFR-2, VEGFR-3, FGFR1, FGFR2, FGFR3, FGFR4, PDGFRA, RET, KIT
	Nintedanib	VEGFR-1, VEGFR-2, VEGFR-3, PDGFRA, PDGFRB, FGFR1, PGFR2, PGFR3, FLT3, LCK, LYN, SRC
	Regorafenib	VEGFR-1, VEGFR-2, VEGFR-3, KIT, PDGFRA, PDGFRB, FGFR1, FGFR2, TEK, DDR2, NTRK1, EPHA2, RAF1, BRAF, MAPK11, FRK, ABL1, RET
	Sorafenib	VEGFR-1, VEGFR-2, VEGFR-3, BRAF, RAF1, FLT3, PDGFRB, KIT, FGFR1, RET
	Tivozanib	VEGFR-1, VEGFR-2, VEGFR-3, KIT, PDGFRB, FLT3, PDGFRA, PTK6, TEK, FGFR1, MET
	Vatalanib	VEGFR-1, VEGFR-2, VEGFR-3, PDGFR, KIT
	Vandetanib	VEGFR-1, VEGFR-2, VEGFR-3, PDGFR, EGFR
	Ponatinib	VEGFR-2, ABL1, BCR, KIT, RET, TEK, FLT3, FGFR1, FGFR2, FGFR3, FGFR4, LCK, SRC, LYN, PDGFRA

^a^ ABL1: Tyrosine-protein kinase ABL1; BCR: breakpoint cluster region protein; BRAF: Serine/threonine-protein kinase B-raf; CSF1R: Macrophage colony-stimulating factor 1 receptor; DDR2: Discoidin domain-containing receptor 2; EPHA2: Ephrin type-A, receptor 2; FGFR1: Fibroblast growth factor receptor 1; FGFR2: Fibroblast growth factor receptor 2; FGFR3: Fibroblast growth factor receptor 3; FGFR4: Fibroblast growth factor receptor 4; FGF1: Fibroblast growth factor 1; FLT3: Receptor-type tyrosine-protein kinase FLT3; FRK: Tyrosine-protein kinase FRK; ITK: Tyrosine-protein kinase ITK/TSK; KIT: Mast/stem cell growth factor receptor Kit; LCK: Tyrosine-protein kinase Lck; LYN: Tyrosine-protein kinase Lyn; MAPK11: Mitogen-activated protein kinase 11; MET: hepatocyte growth factor receptor; NTRK1: High affinity nerve growth factor receptor; PDGFRA: Platelet-derived growth factor receptor alpha; PDGFRB: Platelet-derived growth factor receptor beta; PLGF: Placental-derived growth factor; PTK6: Protein-tyrosine kinase 6; RAF1: RAF, proto-oncogene serine/threonine-protein kinase; RET: Proto-oncogene tyrosine-protein kinase receptor Ret; SH2B3: SH2B adapter protein 3; SRC: Proto-oncogene tyrosine-protein kinase Src; TEK: Angiopoietin-1, receptor; VEGF-A: Vascular endothelial growth factor A; VEGF-B: Vascular endothelial growth factor B; VEGFR-1: Vascular endothelial growth factor receptor 1; VEGFR-2: Vascular endothelial growth factor receptor 2; VEGFR-3: Vascular endothelial growth factor receptor 3.

The VEGF family includes seven members: VEGF-A, VEGF-B, VEGF-C, VEGF-D, VEGF-E, and placenta-derived growth factor (PLGF). VEGF binds to VEGFRs (VEGFR-1 and VEGFR-2) in the signaling pathway. VEGFR-2 is the primary signaling receptor for VEGF binding. The VEGF-A/VEGFR signal axis induces the process of vasculogenesis and angiogenesis. VEGF-C and VEGF-D bind to VEGFR-3, a master regulator of lymphangiogenesis. It is a high affinity that VEGFR-1 can bind with VEGF-A, VEGF-B, and PLGF. As a kinase insert domain receptor, VEGFR-2 is encoded by the human KDR gene. It can bind with VEGF-A, VEGF-C and VEGF-D. (Ahmad and Nawaz, 2022).

The humanized anti-VEGF monoclonal antibody, bevacizumab, binds VEGF with an affinity very similar to that of the original antibody. Bevacizumab binds and neutralizes all human VEGF-A isoforms and bioactive proteolytic fragments, as does its mouse counterpart. (Ferrara et al., 2004). Ramucirumab effectively prevents VEGF ligands from binding and activating the receptor by binding to both soluble and cell surface VEGFR-2. (Sanchez-Gastaldo et al., 2016). Aflibercept, as a decoy receptor, targets VEGF-A, VEGF-B, and PLGF to inhibit angiogenesis. It has a higher affinity for VEGF-A than either VEGFR or bevacizumab. (Ciombor and Berlin, 2014).

Upregulation of alternative angiogenic signaling pathways, including PDGF and fibroblast growth factor (FGF), is a postulated mechanism by which resistance to anti-VEGF therapy may develop. TKIs that inhibit multiple angiogenic pathways may help overcome this resistance by blocking overlapping pathways. Axitinib, sorafenib, sunitinib, cediranib, tivozanib, cabozatinib, vatalanib, vandetanib, pazopanib, nintedanib, lenvatinib, ponatinib, and regorafenib inhibit VEGFR and other targets (Table 1). (Wood et al., 2000; Wedge et al., 2002; Mendel et al., 2003; Wilhelm et al., 2004; Wedge et al., 2005; Nakamura et al., 2006; Patyna et al., 2006; Polverino et al., 2006; Wilhelm et al., 2006; Kumar et al., 2007; Roskoski, 2007; Sonpavde and Hutson, 2007; Hilberg et al., 2008; Hu-Lowe et al., 2008; Wilhelm et al., 2011; Gozgit et al., 2013; King and Lee, 2013)

Since VEGF is widely distributed in other tissues and organs such as the heart, promoting the formation of new blood vessels and the growth of vascular endothelial cells, it also plays a key role in the blood vessels of non-tumor tissues. While acting on the tumor vascular system, VEGFI inevitably inhibits the blood vessels of non-tumor tissues, leading to cardiotoxicity. VEGFI-associated cardiovascular adverse reactions include hypertension, left ventricular systolic dysfunction, and QT interval prolongation, along with arterial and venous thromboembolism, heart failure, and arrhythmia (Dobbin et al., 2021). However, previous literature on the subject rarely discusses that VEGFI could induce aortic dissection (AD).

**FIGURE 2:**
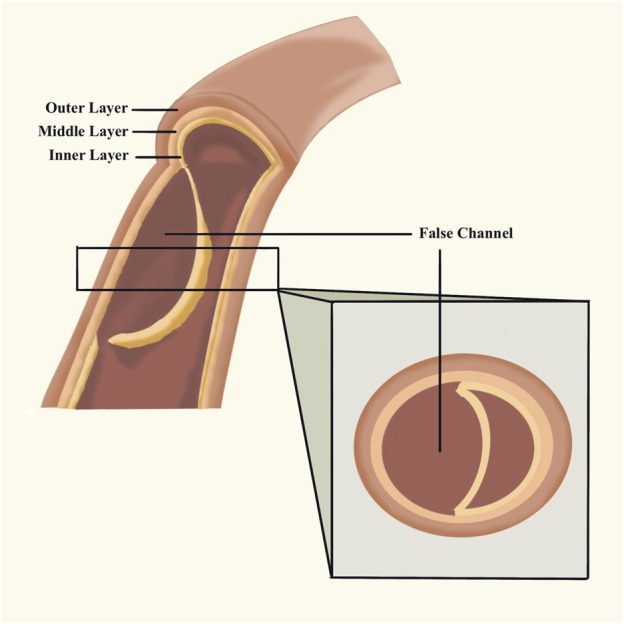
Aortic dissection. The blood stream reenters the aorta through another tear in the inner layer and forms thrombus the false channel.

The aorta is composed of the inner, middle, and outer layers. The elastic muscle in the layer protects the wall against blood pressure. The existence of physiological conditions and use of VEGFI might weaken the media. A crevasse is commonly caused by blood pumping against the weakened section of the inner layer. Blood moves through the break in the inner wall, separating it from the middle. Consequently, there is a septum between the true aortic channel and the false channel, called aortic dissection. Due to the tear in the inner layer, the blood may break through the outer layer of the aortic wall, causing a life-threatening problem, or it may reenter the aorta through another tear in the inner layer. As time goes on, there may be a thrombus in the false channel. (Figure 2). There are the Stanford type and the Debakey type. The classification of AD is Stanford type A if the ascending aorta is involved (historically DeBakey type I and type II), and Stanford type B if the descending aorta is involved (historically DeBakey type IIIa or type IIIb). (Carrel et al., 2023). Debakey type I begins in the ascending aorta and extends distally throughout the remaining aorta. Debakey type II is limited to the ascending aorta. Debakey type IIIa describes a limited dissection to the thoracic aorta, whereas type IIIb describes more distal extension into the abdominal aorta. (Debakey et al., 1965).

AD is a critical emergency disease in cardiovascular disease, with acute onset, rapid progression, high mortality rate, and easy to be misdiagnosed and missed. Although analysis of the relation between AD and VEGFI based on the databases has been reported (Oshima et al., 2017; Cheng et al., 2021; Dörks et al., 2021; Wang et al., 2021), the current literature lacks a comprehensive review of containing wilder population in which aortic dissection occurs. To provide general insight into VEGFI and AD, we reviewed the case reports and discuss the pathophysiology, risk factors, diagnosis, treatment, and future directions of innovation.

## 2 Case reports collection

The search was conducted in PubMed and CNKI (China National Knowledge Infrastructure) from inception to 28 April 2022. The terms for the search strategy were “aortic dissection” and “antiangiogenic”, “angiogenesis inhibitors”, “vascular endothelial growth factor”, “VEGF”, “VEGFR”, “sunitinib”, “axitinib”, “sorafenib”, “anlotinib”, “apatinib”, “lenvatinib”, “vandetanib”, “pazopanib”, “cabozantinib”, “nintedanib”, “ponatinib”, “regorafenib”, “vatalanib”, “bevacizumab”, “ramucirumab”, “aflibercept”. The resulting articles were screened for case reports.

**FIGURE 3:**
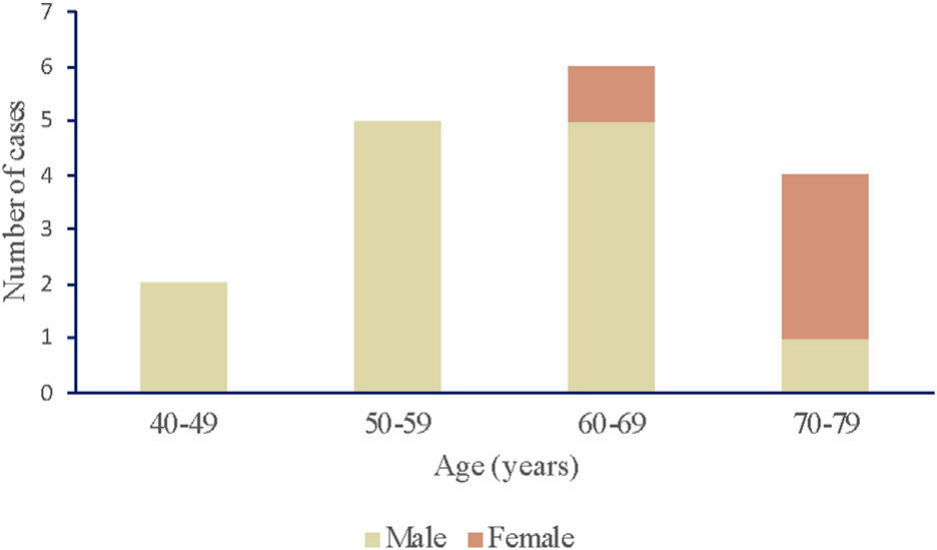
The distribution of cases by age and sex.

**FIGURE 4:**
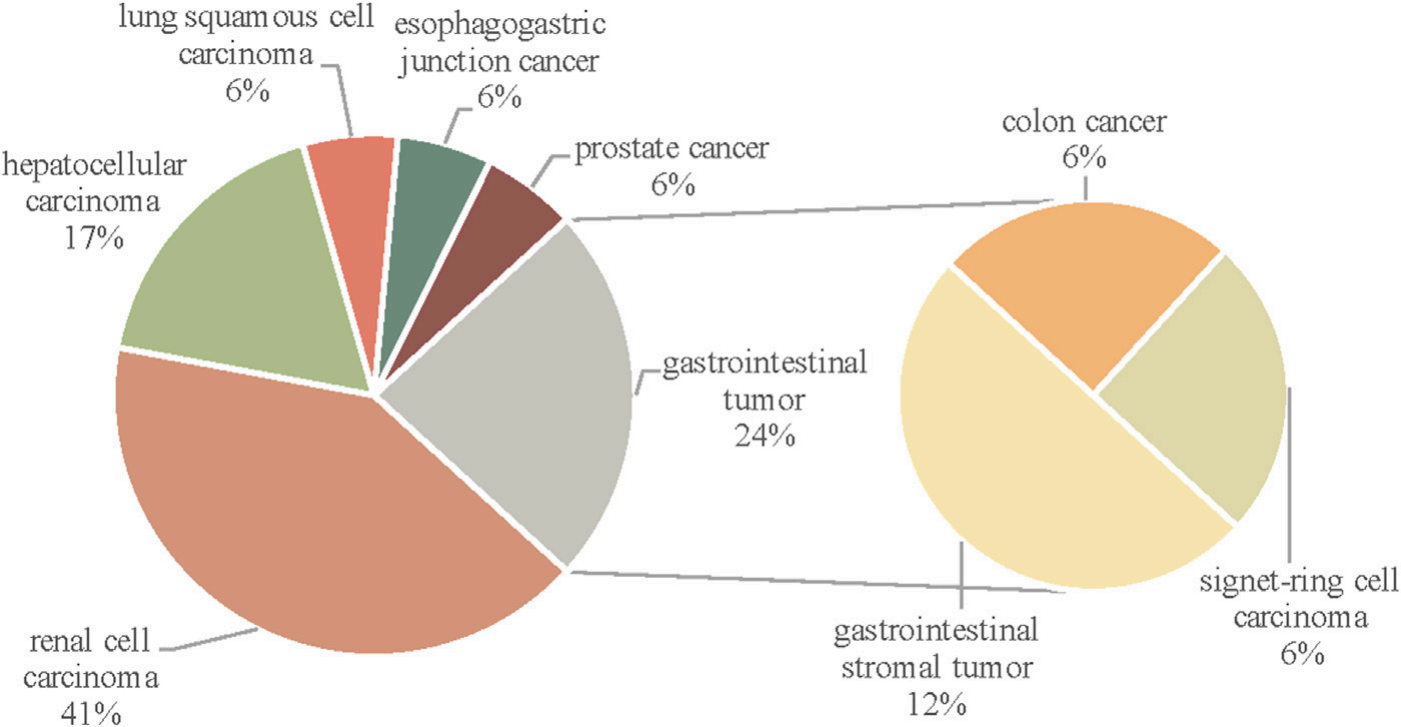
The distribution of cases by tumor type.

**FIGURE 5:**
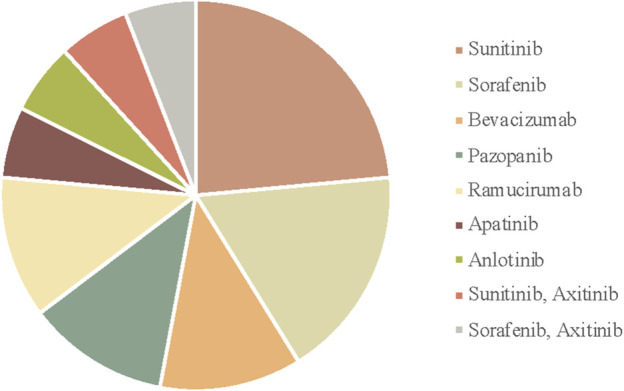
The distribution of cases by VEGFI.

In total, seventeen articles were selected for this study. Information was individually extracted from each article, including gender, age, type of tumors, VEGFI drug and duration, types of AD, history, adverse reaction, treatment for AD, and outcome. Findings are summarized in Table 2 and Figures 3–5.

**TABLE 2:** Information on patients included in the case reports.

Case reports	Year	Age	Sex	Past history	Tumor type	Tumor progression	VEGFI	Duration	ADR during VEGFI treatment	AD type	Treatment	Drugs	Outcome
1 Aragon-Ching et al. (2008)	2008	70	male	hypertension	prostate cancer	bone metastatic	bevacizumab	28 cycles	grade 3 hypertension	N/A	antihypertensive medication	C, hydralazine	recovered
2 Edeline et al. (2009)	2009	58	male	hypertension	renal cell carcinoma	lung metastasis	sunitinib	4 cycles	thoracic pain	Stanford type B	N/A	N/A	N/A
3 Carles (2010)	2010	77	female	N/A	renal cell carcinoma	stage IV	sorafenib	3 cycles	epigastric pain, fatigue	N/A	antihypertensive medication, analgesic measures	lanetalol, B, A (ACEI), D	recovered
4 Formiga and Fanelli (2014)	2014	68	male	smoking	renal cell carcinoma	metastatic	sunitinib	2 cycles and 20 days	thoracic pain, hypertension	Stanford type B	antihypertensive medication	metoprolol, sodium nitroprusside	recovered
5 Kurtz et al. (2014)	2014	48	male	hypertension	renal cell carcinoma	N/A	pazopanib	4 years and 8 months pazopanib and lapatinib	Hypertension, proteinuria, grade 1 hypopigmentation of the skin, grade 3 stomatitis, grade 3 thrombocytopenia, grade 1 thyroid dysfunction, no thoracic pain	Stanford type A	N/A	N/A	died
6 Niwa et al. (2015)	2015	51	male	N/A	renal cell carcinoma	lung metastasis	sunitinib, axitinib	2 cycles sunitinib, 10 days axitinib	grade 3 palmar-plantar erythrodysesthesia, midsternal pain	Stanford type A	reconstruction of the aorta	N/A	N/A
7 Koda et al. (2016)	2016	77	female	N/A	gastrointestinal tumor	peritoneal dissemination and lung metastasis	bevacizumab	8 cycles	aortoesophageal fistula rupture	Stanford type B	endovascular chest stent grafting	N/A	recovered
8 Hatem et al. (2017)	2017	63	female	no history of hypertension, cardiovascular disease or risk factors for coronary artery disease	gastrointestinal tumor	N/A	sunitinib	21 days	cephalalgia, hot flashes, nausea, oedema	N/A	replacement of the aorta	N/A	recovered
9 Xu et al. (2017)	2017	49	male	hypertension	hepatocellular carcinoma	N/A	sorafenib	4 months	N/A	N/A	endovascular chest stent grafting	N/A	N/A
10 Atsunori et al. (2018)	2018	65	male	N/A	hepatocellular carcinoma	N/A	sorafenib	2 weeks	hypertension	Stanford type A	replacement of the aorta, antihypertensive medication	A (ARB)	recovered
11 Takada et al. (2018)	2018	66	male	N/A	renal cell carcinoma	lung metastasis	sorafenib, axitinib	27 months sorafenib, 51 months axitinib	grade 2 liver dysfunction, hypertension, proteinuria, cardiac dysfunction, cardiomyopathy, back pain, fatigue, palpitation, aortic regurgitation	Stanford type A	antihypertensive medication	candesartan, amlodipine, azelnidipine, loxoprofen, carvedilol	recovered
12 Toru et al. (2018)	2018	66	male	N/A	gastrointestinal tumor	hepatic metastasis	sunitinib	6 cycles	hypertension	Stanford type A	antihypertensive medication	N/A	N/A
13 Fubo and Gong (2019)	2019	63	male	no history of hypertension, cardiovascular disease or risk factors for coronary artery disease	hepatocellular carcinoma	intrahepatic metastasis	apatinib	4 months	hypertension, xiphoid process pain, dizziness	N/A	antihypertensive medication, analgesic measures	N/A	N/A
14 Zenoni et al. (2019)	2019	70s	female	systemic arterial hypertension; carotid atherosclerosis, cerebellar stroke	gastrointestinal tumor	N/A	ramucirumab	1 day	cold sweating, hypotension with lipothymia	Stanford type A	analgesic measures, sedative-hypnotics	morphine hydrochloride, midazolam, haloperidol	died
15 Jiang et al. (2020)	2020	58	male	nephrolithiasis	lung squamous cell carcinoma	left hilar lymph node metastasis	anlotinib	4 cycles	grade 2 fatigue, grade 2 palmar-plantar erythrodysesthesia, grade 1 oral mucositis, and Grade 2 hypertriglyceridemia, back pain	Debakey type III	endovascular chest stent grafting, antihypertensive medication, Anticoagulants	nitroglycerin	recovered
16 Nikai et al. (2020)	2020	59	male	N/A	esophagogastric junction cancer	para-aortic lymph node metastasis, bone metastases	ramucirumab	3 cycles	neck pain	Stanford type A	N/A	N/A	died
17 Ting and Lo (2021)	2021	59	male	history of peptic ulcer disease	renal cell carcinoma	metastasis	pazopanib	3 months	hypertension, hepatotoxicity, thyroid dysfunction, epigastric pain	Stanford type B	antihypertensive medication	labetalol, carvedilol, nifedipine	recovered

^a^ N/A: not available.

The review of the literature identified seventeen eligible case reports with VEGFI related to AD (Aragon-Ching et al., 2008; Edeline et al., 2009; Carles, 2010; Formiga and Fanelli, 2014; Kurtz et al., 2014; Niwa et al., 2015; Koda et al., 2016; Hatem et al., 2017; Xu et al., 2017; Atsunori et al., 2018; Takada et al., 2018; Toru et al., 2018; Fubo and Gong, 2019; Zenoni et al., 2019; Jiang et al., 2020; Nikai et al., 2020; Ting and Lo, 2021). Thirteen of the seventeen patients are male, and four patients are female. Of the seventeen case reports included in our analysis, seven were conducted in patients with renal cell carcinoma; four were in patients with gastrointestinal tumor (two cases in gastrointestinal stromal tumor [Hatem et al., 2017; Toru et al., 2018), one case in colon cancer (Koda et al., 2016), one case in signet-ring cell carcinoma (Zenoni et al., 2019)]; three results related to patients with hepatocellular carcinoma; one for patients with esophagogastric junction cancer; one was in patients with lung squamous cell carcinoma; one was in patients with prostate cancer.

The patients using VEGFI including sunitinib, sorafenib, pazopanib, axitinib, apatinib, anlotinib, bevacizumab, and ramucirumab are from 48 to 70 s years old. The occurrence of aortic dissection ranges from 1 day to 6 years after the application of VEGFI, which shows the possibility of aortic dissection at any time with VEGFI.

Among the screened cases, eleven case reports (Table 2) described adverse reactions and most of them showed increased blood pressure, besides proteinuria, hypopigmentation of the skin, thyroid dysfunction, stomatitis, thrombocytopenia, palmar-plantar erythrodysesthesia, aortoesophageal fistula rupture, cephalalgia, hot flashes, nausea, oedema, liver dysfunction, cardiac dysfunction, cardiomyopathy, fatigue, and hypertriglyceridemia. The patients were administered antihypertensive drugs, besides sedative hypnotics, analgesics, and anticoagulants.

The five patients that reported AD had pre-existing hypertension before VEGFI medication, while two had no hypertension. Of the seventeen cases, seven cases were Stanford type A, four in Stanford type B, one in DeBakey type III [equivalent to Stanford type B (Surgeon, 2017)], and five were not mentioned in the article. Among the non-mentioned cases, it was inferred from the reports that three (Aragon-Ching et al., 2008; Carles, 2010; Xu et al., 2017) were Stanford type B (Ince and Nienaber, 2007).

The three patients who died due to AD were all Stanford type A, and treated with ramucirumab (two cases) and pazopanib (one case). The age distribution of patients who died was the 40 s, 50 s, and 70 s, indicating that age was not a risk factor for death. Among them were two male and one female patient. Notably, two patients who died had a history of hypertension. We supposed that the physical condition with high blood pressure made the patients’ recovery difficult.

Among the patients with AD, there were significantly more men than women. According to the age analysis of the patients in these case reports, although the ages ranged from 48 to 77 years old, the majority (*n* = 14, 82.4%) of patients were concentrated in the 58 to 67-year-old range.

Overall, across the case reports, the treatment for AD included reconstruction and replacement of the aorta, endovascular chest stent grafting, and drug therapy.

## 3 Discussion

### 3.1 Pathology

The etiology and mechanism of aortic dissection have been the subject of research and some progress has been made, but the specific pathogenesis of aortic dissection has not yet been outlined conclusively. Abnormal phenotypic transformation and apoptosis of vascular smooth muscle cells, abnormal degradation of extracellular matrix, endothelial dysfunction, and immune cell infiltration have been implicated in the pathogenesis of aortic dissection (Yin et al., 2022).

In vasa vasorum, the VEGF signal pathway is related to the activation of the phosphatidylinositol-3-kinase-AKT signaling pathway. Inhibition of the pathway might result in overexpression of matrix metalloproteinase 9 (MMP9), which leads to extracellular matrix degradation. (Takada et al., 2018). Besides the previous point on the overexpression of MMP9, the inhibition of the VEGF signal pathway might induce the increase of NO, which leads to SMC apoptosis.

The driving mechanism of aortic dissection is aortic intima tear and cystic medial necrosis. First, the existence of some underlying diseases increases blood cholesterol and glucose levels, which leads to atherosclerosis. These changes negatively affect collagen formation and connective tissue strength, leading to increased vascular fragility and increased blood pressure. (Kurtz et al., 2014). Meanwhile, VEGFI may cause increased stiffness in arteries, making them more subject to dissection. Then, VEGFI such as sunitinib (Edeline et al., 2009), sorafenib (Carles, 2010), and pazopanib (Kurtz et al., 2014) target VEGF and platelet-derived growth factor receptors, interfering with the normal function of these factors. The damaged vascular endothelium cannot regenerate and heal normally, and AD is formed eventually.

VEGFI also impairs vasodilation and increases vasoconstriction. It reduces NO and prostacyclin to weaken vasodilation and increases the secretion of endothelin-1 from vascular endothelial cells to enhance vasoconstriction. Another proposed mechanism is that VEGF helps regulate the sympathetic innervation of blood vessels (Hatem et al., 2017).

### 3.2 Risk factors

Aortic dissection generally occurs in patients who have gotten hypertension, atherosclerosis, diabetes, and Marfan syndrome. It is considered that hypertension is the most common predisposing factor for aortic dissection (Ince and Nienaber, 2007). However, in two cases, hypertension had not been observed before VEGFI therapy (Hatem et al., 2017; Fubo and Gong, 2019).

Oshima et al. (Oshima et al., 2017) have outlined that the use of VEGFI had a comparatively higher adjusted odds ratio than hypertension. However, Dörks (Dörks et al., 2021) suggested that the dose-rising of VEGFI was related to increased blood pressure. Pre-existing hypertension is not an inevitable factor for VEGFI causing AD. The increase in blood pressure after the medication is more critical. Together, the combination of pre-existing hypertension and increased blood pressure after VEGFI causes the risk of AD.

One case (Toru et al., 2018) had a history of vascular calcification. It could be considered that AD was caused by two reasons: (a) hypertension caused by sunitinib; and (b) vascular fragility caused by vascular calcification and sunitinib.

The main parallel between Cheng et al. (Cheng et al., 2021) and our studies indicate that the most common underlying risk factor reported in the case series is hypertension. Other cases have also been reported without preexisting hypertension. Both case series reported axitinib, bevacizumab, pazopanib, ramucirumab, sorafenib, and sunitinib related to AD. In the study by Cheng et al. 44% (105/240) of cases were reported in female patients. Cabozantinib, lenvatinib, ponatinib, regorafenib, vandetanib, and ziv-aflibercept were reported. Among our screened cases, thirteen of the seventeen patients were male, and four patients were female. Apatinib and anlotinib were reported.

### 3.3 Diagnosis

The main complaint of AD patients is tear-like or knife-like persistent and unbearable sharp pain. The location of the pain is related to the rupture and progression of AD. Stanford type A presents with chest pain or back pain, and Stanford type B presents with back pain or abdominal pain. The two pain areas may also overlap. The possibility of AD should be suspected in patients with severe chest and back pain with high-risk medical history and signs. Other than that, migrating pain may indicate the progression of dissection. If the patient has lower extremity pain, it may indicate that the dissection may involve the iliac or femoral arteries. However, some patients may also have no pain symptoms (Surgeon, 2017). Among the patients mentioned in this review, a patient (Kurtz et al., 2014) with Stanford type A complained of no chest pain. In addition to pain, AD could lead to cardiac complications and other adverse effects of organ perfusion. Hypotension in some patients may be related to cardiac tamponade or dissection involving the brachiocephalic artery.

The initial examination usually uses imaging, which aims to comprehensively evaluate the whole aorta, including the scope and shape of AD involvement, the diameter of the aorta in different parts, the involvement of the aortic valve and its branches, the relationship with surrounding tissues, and other AD relevant manifestations such as pericardial effusion, pleural effusion, and organ ischemia (Surgeon, 2017). Computed tomography angiography (CTA) is the first-choice imaging method for the diagnosis of AD, and magnetic resonance imaging (MRI) can be used when CTA cannot be performed. Although the diagnostic accuracy of echocardiography for AD is slightly lower than that of CT and MRI, it can be used for preoperative, intraoperative, and postoperative evaluation of patients with various conditions because of its strong portability.

In addition, the D-dimer test is used as the diagnosis and differential diagnosis of AD. Routine laboratory inspection items cover blood routine and blood type, urine routine, biochemical complete set, blood gas analysis, hepatitis B and other infectious disease screening, myocardial enzymes, and myocardial markers, myoglobin, coagulation five items inspection. In the acute phase, leukocytosis with an increased proportion of neutrophils and an increased erythrocyte sedimentation rate is common. Another biomarker is plasma C-reactive protein, which can reflect the inflammatory activity.

AD needs to be differentiated from acute myocardial infarction, acute abdomen, acute pulmonary embolism, acute pericarditis, and pneumothorax to avoid misdiagnosis and delay the disease. The doctors who initially treat patients should improve understanding of the AD and correctly analyze the condition. Patients with abdominal pain symptoms and previous abdominal diseases should not be missed diagnosis. The clinical manifestations of AD are complex and lack specificity, and chest pain, abdominal pain, and back pain are common. It is especially important to choose the correct auxiliary examination, such as CTA. Blood pressure monitoring and control should be routinely performed (Fubo and Gong, 2019).

### 3.4 Treatment

Pharmacotherapy, surgery, and endoluminal intervention are mainly used in the treatment of AD. The general purpose of drug therapy is to relieve pain and reduce the rate of ejection from the left ventricle and systolic blood pressure. Morphine is commonly used in analgesics. Drugs controlling blood pressure are A (angiotensin converting enzyme inhibitors (ACEI), angiotensin receptor blockers (ARB)), B (β-receptor blockers), C (calcium channel clockers), and D (diuretic). B is recommended in AD, which includes metoprolol and labetalol. While there are contraindications to B, C is used. During the course of treatment, adjuvant medication including sedative-hypnotics and anticoagulants help improve patients’ compliance and prognosis.

Stanford type A is generally considered surgery which is artificial vascular grafting, replacement, and reconstruction, removing the aortic segment where the intimal tear is located. Age is not a contraindication to surgery, but for patients of advanced age, the condition of other organs should be comprehensively evaluated, such as type A in a persistent coma that is not suitable for surgery.

Patients with Stanford type B are treated with endovascular intervention, known as endovascular repair of thoracic aortic dissection (TEVAR). This treatment method inserts a stent graft into the true lumen and blocks the primary rupture of the dissection, so that the blood in the false lumen loses communication, effectively reducing the pressure of the false lumen and reducing the risk of aortic rupture.

### 3.5 Future direction

This review provides doctors with a working direction for the long-term recording of cardiac function in cancer patients and suggests the key points of diagnosis and treatment of AD. Patients using VEGFI must be aware of symptoms relating to pain and the fluctuation of blood pressure.

### 3.6 Limitations

Due to the low probability of AD in patients, there are only a few case reports and no randomized clinical trials to provide a statistical basis. Case reports can also have limitations in terms of under-reporting and non-reporting adverse reactions. Thus, our results only indicate an increased risk, which must be confirmed in prospective controlled trials.

## 4 Conclusion

Vascular endothelial growth factor inhibitors are related to aortic dissection. AD must be diagnosed early and treated aggressively in good time, or else it can be fatal. Although current literature lacks clear statistical evidence on the population, we offer these points to encourage further confirmation of the care for these patients.
